# Colorectal liver metastases: making the unresectable resectable using irreversible electroporation for microscopic positive margins – a case report

**DOI:** 10.1186/s12885-015-1279-9

**Published:** 2015-04-12

**Authors:** Hans F Schoellhammer, Bryan Goldner, Shaila J Merchant, Jonathan Kessler, Yuman Fong, Singh Gagandeep

**Affiliations:** 1 Division of Surgical Oncology, Department of Surgery, City of Hope Comprehensive Cancer Center, 1500 E. Duarte Road, Duarte, CA 91010 USA; 2 Division of Interventional Radiology, Department of Radiology, City of Hope Comprehensive Cancer Center, 1500 E. Duarte Road, Duarte, CA 91010 USA

**Keywords:** Irreversible electroporation, Colorectal liver metastases, Margin ablation, IRE, CRLM, Liver resection, Metastasectomy

## Abstract

**Background:**

Irreversible electroporation (IRE) is a non-thermal injury tissue ablation technique that uses electrical pulses to cause cell death. IRE damages the endothelial cells of blood vessels; however these cells re-grow, and thus IRE does not result in permanent damage to blood vessels. We report the novel use of IRE for ablation of microscopically positive margins after resection of colorectal liver metastases (CRLM) impinging on hepatic veins.

**Case presentation:**

A 68-year-old female was found to have colon cancer and synchronous bilateral unresectable liver metastases. Chemotherapy with FOLFOX and cetuximab was initiated, with subsequent conversion to resectability of the CRLM. The patient underwent colectomy followed by right liver posterior sectionectomy with wedge resection of segment 5. Resection of tumor impinging on the left and middle hepatic veins would have required left hepatectomy, with insufficient remnant liver volume. The CRLM were meticulously dissected off the hepatic veins leaving a microscopically positive margin, and IRE was then used for margin ablation, leaving intact hepatic veins and venous blood flow. The patient is alive and without recurrent disease now 30 months after resection. Herein we review the IRE technology and its use in ablation of liver metastases.

**Conclusions:**

Use of IRE margin ablation for microscopically-positive CRLM resection may lead to long-term patient survival; further prospective randomized trials are needed to confirm this finding.

## Background

Resection of colorectal liver metastases (CRLM) has been well described, with reports of five-year survival up to 58% after complete resection of disease [1]. The overarching goals of liver resection for CRLM are to obtain tumor-free margins and a functioning liver remnant with intact portal venous and arterial inflow, venous outflow, and biliary-enteric drainage [2,3]. An operation should be undertaken with the intent of a margin-negative resection, as patients with a positive resection margin have significantly increased risk for local recurrence as well as significantly decreased overall survival compared with margin-negative patients [4]. The extent of liver resection, and the success of a margin-negative resection, may be limited by concomitant intrinsic liver disease, chemotherapy-induced liver damage, and the anatomic location of the metastases. Tumors adjacent to major hepatic blood vessels may pose a challenge to resection if, in taking the tumor and associated blood vessel, an inadequate amount of liver is left behind to support hepatic function post-operatively.

In the past, tumor adjacent to major hepatic blood vessels (*e.g.*, hepatic vein, portal vein) has been a relative contraindication to surgery if tumor-free margins are unable to be achieved while maintaining a sufficient liver remnant; however, with the use of irreversible electroporation (IRE), a relatively new ablation technique, in combination with hepatic resection, tumors may be safely treated while preserving the hepatic vasculature. Irreversible electroporation is a non-thermal injury tissue ablation technique that uses electrical pulses of short duration to permanently create defects in cell membranes leading to irreversible permeabilization and ultimately cell death [5]. Vessels adjacent to the zone of ablation do not cause a heat sink effect, as may be seen in radiofrequency or microwave ablation. Furthermore, IRE has a very low incidence of collateral damage to adjacent structures, making it possible to ablate tumors adjacent to major hepatic vessels [6]. In this report we describe a novel technique of hepatic resection followed by margin ablation using IRE as a treatment for CRLM near major vascular structures, resulting in long-term and durable patient survival.

## Case presentation

**Figure 1:**
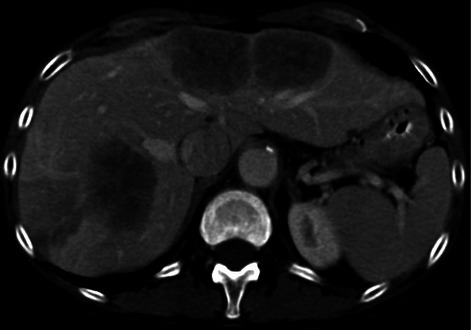
On initial presentation the patient was found to have multiple hypodense hepatic metastases in segments 2 and 4A as well as segments 5-7 and the burden of disease was deemed not resectable.

The patient is a 68-year-old female who sought medical care at an outside facility after passing bright red blood per rectum. Laboratory evaluation demonstrated anemia. The patient was otherwise without other complaints at the time of presentation, denying nausea, vomiting, change in bowel habits, jaundice, decreased appetite and early satiety. The patient underwent a diagnostic colonoscopy which demonstrated a friable ulcerated circumferential mass in the mid-transverse colon, causing 50% occlusion of the lumen; biopsy of the mass demonstrated colonic adenocarcinoma that was *KRAS* wild-type. The patient underwent full staging with CT scans of the chest, abdomen, and pelvis, which confirmed the mass in the mid-transverse colon, as well as multiple large hypodense hepatic lesions located in segments 2, 4A, 5, 6, 7 (Figure 1). This workup was performed at the outside facility, including the performance of CT-guided liver biopsy which confirmed metastatic adenocarcinoma. Liver function tests and coagulation parameters were all normal at the time of presentation.

The patient was referred to medical oncology and began chemotherapy with fluorouracil, oxaliplatin, and leucovorin (FOLFOX) with cetuximab. The patient underwent seven cycles of chemotherapy over three months and was subsequently referred to our institution for evaluation.

**Figure 2:**
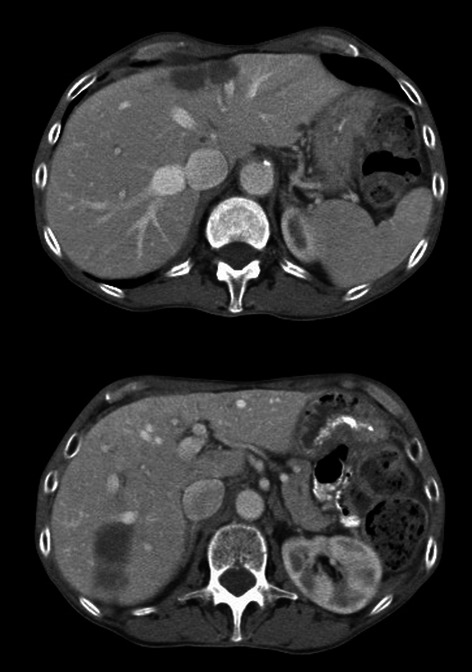
Appearance of hepatic metastases in segments 2 and 4A (top) and segments 5-7 (bottom) after treatment with FOLFOX chemotherapy with cetuximab. The disease burden has decreased and the patient has been converted to resectable status.

Upon review of restaging CT and PET/CT scans obtained during and after chemotherapy, the patient was noted to have decrease in size of both the primary colonic lesion as well as the liver metastases, without evidence of new foci of metastatic disease (Figure 2). Both the primary colon cancer and hepatic metastases were deemed to be resectable, with the proposed surgical plan being an extended right colectomy with a right posterior sectionectomy and wedge resections of the segment 2, 4A, and 5 lesions.

The patient was taken to the operating room and underwent a right colectomy with ileocolic anastomosis without incident and attention was then turned to the liver. Intra-operative hepatic ultrasound revealed three multi-lobulated lesions within segments 6 and 7, a small segment 5 lesion, as well as a segment 2 and 4A lesion between the middle and left hepatic veins. The metastases were resectable, and the liver, while appearing to have some damage as a result of chemotherapy, was felt to have adequate volume for a functioning liver remnant.

The segment 5 lesion was wedged out in a standard fashion using electrocautery and hemostatic clips. This was a margin-negative resection despite its close proximity to the portal venous system. A right posterior sectionectomy was performed removing segments 6 and 7, along with a cholecystectomy. The right hepatic vein as well as the right posterior portal pedicles, and a few tertiary branches of the right anterior portal vein, were seen entering the tumor and were taken. Intraoperative pathologic assessment of this specimen reported negative margins (3-4 mm).

**Figure 3:**
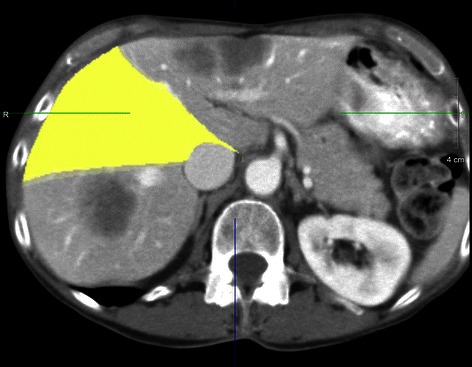
Volumetrics of highlighted segments 5/8 demonstrate a future liver remnant of approximately 30%.

**Figure 4:**
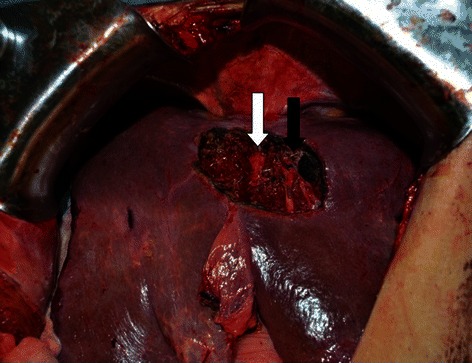
Intra-operative photograph after resection of tumor from segments 2 and 4A with likely microscopically positive margin after tumor was carefully dissected off of the middle hepatic (white arrow) and left hepatic veins (black arrow).

Attention was then turned to the lesions in segments 2 and 4A. A formal left hepatectomy would have allowed for a margin-negative resection; however the end result would have resulted in less than 30% liver remnant in an already chemotherapy-treated liver. Based on pre-operative volumetric data, the right anterior section constituted approximately 30% future liver remnant (Figure 3), and following a segment 5 wedge resection this volume was further reduced. It was decided to dissect out the tumor, lifting it off of the underlying hepatic veins (Figure 4). No attempt was made to obtain negative margins because of extreme proximity to the hepatic veins, and thus the tumor was meticulously dissected off of the veins with the understanding that microscopic disease would be left on the vessels. Following completion of tumor excision, normal flow was seen in the left and middle hepatic veins on ultrasound.

Given the microscopic positive margins at the middle and left hepatic veins, we decided to ablate the vein margins using IRE (NanoKnife System, AngioDynamics, Latham, NY). The resection bed of segments 2 and 4A was ablated using electroporation needle pairs placed 1.5 cm apart and 1.5 cm deep into the hepatic parenchyma that remained just adjacent to the hepatic veins. The needle pairs were placed parallel to the veins and the covered with a free flap of omental fat that had been harvested to add extra tissue through which the electrical current would travel such that the positive surfaces of the veins would be treated. Treatments were performed with settings of 3000 V using 100 microsecond pulses for a total of 90 pulses between each needle pair. A total of seven overlapping ablations were performed across the entire raw surface area. The omental fat pad was discarded, and following the completion of IRE, adequate flow was again confirmed through the left and middle hepatic veins without evidence of thrombosis. The patient tolerated the procedure well and her post-operative course was uneventful. The patient was discharged home on post-operative day 9 in good condition with normal liver function.

**Figure 5:**
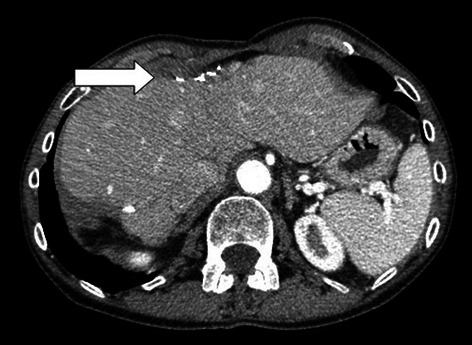
Appearance of the liver remnant 18 months after resection; there is no evidence of recurrent disease at the site of margin ablation in segments 2/4A (arrow) or in the remainder of the liver, the middle and left hepatic veins are patent, and the liver remnant has undergone hypertrophy.

Pathology of the resected right colon demonstrated well-to-moderately differentiated adenocarcinoma invading through the muscularis propria to the subserosal tissue. All margins were negative and 0/43 regional lymph nodes were positive for carcinoma. The final pathology of the resected right posterior section demonstrated three masses, the largest of which was 3 cm, and negative margins. Pathologic result of the segment 2/4A resection specimen demonstrated a 3.6 cm mass with viable carcinoma present at the inked margin of resection, as had been anticipated, and which had been ablated by IRE. The patient now continues to be under active surveillance and has shown no demonstrable evidence of recurrence along the ablated margin 30 months after the operation (Figure 5).

## Discussion

Resection of CRLM has been widely described, and survival has been reported to range anywhere from 25-70% following surgical resection [2,7,8]. Previously-held traditional resection principles, such as obtaining 1 cm margins [9] have changed, and eligibility for curative resection now depends on the ability to obtain negative margins on all lesions with an adequate liver remnant [3,10].

Irreversible electroporation is a non-thermal ablation technique whereby electrical pulses delivered to tissue between two or more probes cause the creation of permanent cell membrane nanopores, with subsequent breakdown of cell membranes and death by apoptosis [5]. Notably, after IRE the extracellular matrix is left intact, which allows for the preservation of blood vessels and bile ducts [11]; this quality makes IRE an attractive adjunctive treatment in the management of tumors abutting major vascular and biliary structures by limiting collateral damage done to these critical structures during ablation. In addition, thermal ablation modalities that depend on heating the tissue to be ablated to 60°C for cell death may fail to reach the target temperature in areas adjacent to large vessels due to a heat sink effect with risk for incomplete ablation and recurrence [6]; this is not the case with the use of IRE near large vessels.

While response rates and long-term survival data from large prospective clinical trials using IRE are still awaited, multiple previous case report series have detailed the use of IRE for the treatment of both metastatic and primary carcinomas of the liver (Table 1), and the procedure has been found to be safe and efficacious. Cannon and colleagues prospectively analyzed outcomes of 44 patients with hepatic tumors (20 patients with CRLM, 14 hepatocellular carcinoma [HCC], and 10 other metastasis) who underwent 48 IRE procedures [12]. In all 48 of the IRE procedures the tumors were located in close proximity to major vascular or biliary structures or to adjacent organs. Nine adverse events occurred in five patients, all of which resolved within 30 days, and no deaths, biliary stricture, or portal vein thromboses were reported. Local recurrence free survival was reported at 3, 6, and 12 months to be 97.4%, 94.6%, and 59.5%, respectively, and was 100%, 100%, and 98% for lesions less than 3 cm. The authors concluded that IRE is safe for use in proximity to vital structures. Similarly, Kingham *et al.* reported their series of IRE in patients with perivascular hepatic tumors not amenable to thermal ablation due to tumor location [13]. Twenty-eight patients underwent 65 IRE treatments, and the median tumor size was 1 cm (range 0.5-5 cm). The authors reported one intra-operative arrhythmia and one post-operative portal vein thrombosis, with an overall morbidity of 3% and no mortalities. With a median follow-up time of six months, one tumor was found to have persistent disease, and three tumors had undergone local recurrence. The authors concluded that IRE is safe in the treatment of perivascular hepatic tumors. Cheung and colleagues reported on eleven patients with HCC who underwent treatment of 18 HCC lesions with IRE with a median follow-up time of 18 months [14]. Of the 18 tumors, 72% were ablated, with 93% success for tumors ≤3 cm. No serious complications were reported, although four patients developed transient urinary retention and seven developed post-procedure pain. The authors reported a local recurrence-free period of 18 ± 4 months and concluded that IRE is not only feasible but safe for the ablation of HCC.

**Table 1:** **Selected reports of irreversible electroporation (IRE) in the treatment of hepatic metastases**

Series	Year	Number of patients	Number of IRE procedures	Complications	Follow-up duration	Recurrence-free survival
Cannon et al. [12]	2013	44	48	9	NR	94.6% 6-mo. 59.5% 12-mo.
Kingham et al. [13]	2012	28	65	2	Median 6 mo	NR
Cheung et al. [14]	2013	11	18	11	Mean 18 mo (14-24)	18 ± 4 mo.

NR = not recorded.

The role of IRE in the spectrum of treatment for tumors metastatic to the liver currently is in evolution and will continue to be refined with time. Currently it appears that the role of IRE will be for local ablation of unresectable masses adjacent to major vascular or biliary structures or for margin ablation to facilitate extended resections, and at the present time IRE will not replace the need for systemic neoadjuvant or adjuvant chemotherapy. It is also currently unclear if disease requiring the use of both liver resection and ablation with IRE indicates more aggressive disease biology, although it is certain that patients with positive surgical margins after resection of CRLM will have worse biology of disease with decreased outcomes and survival [15].

Herein we describe the use of IRE to treat resection margins when the likelihood of positive margins is significant, and the use of IRE in this fashion allows completion of major liver resections in one stage as opposed to the use of a two-stage operation with portal vein embolization. In our case the multiple areas of metastatic disease and disease abutting major vascular structures were impediments to performing a traditional metastasectomy with negative margins. Our resection left microscopic disease behind at the margin abutting the vasculature; however the addition of IRE allowed for ablation of this tissue and destruction of the remaining microscopic disease, thus allowing for complete eradication of the patient’s metastatic disease with preserved hepatic venous flow, an adequately functioning liver remnant, and long-term survival benefit.

## Conclusion

Large prospective trials studying the use of IRE for ablation of unresectable hepatic disease are ongoing; however long-term outcomes data on the use of IRE are currently lacking. We present a novel technique that combines liver resection with ablation of surgical margins using IRE with the intent to eradicate all disease in an area where use of standard microwave or radiofrequency ablation would have resulted in damage to the hepatic veins and a potentially disastrous patient outcome. We believe this technique is especially useful in those patients with poor hepatic reserve, tumors abutting major vasculature or bile ducts, and in those patients with large tumor burdens, and may offer patients the possibility of long-term disease-free survival.

## Consent

Written informed consent was obtained from the patient for publication of this Case Report and any accompanying images. A copy of the written consent is available for review by the Editor of this journal.

## References

[1] Abdalla EK, Vauthey JN, Ellis LM, Ellis V, Pollock R, Broglio KR (2004). Recurrence and outcomes following hepatic resection, radiofrequency ablation, and combined resection/ablation for colorectal liver metastases. Ann Surg.

[2] Aloia TA, Vauthey JN, Loyer EM, Ribero D, Pawlik TM, Wei SH (2006). Solitary colorectal liver metastasis: resection determines outcome. Arch Surg.

[3] Malafosse R, Penna C, Sa Cunha A, Nordlinger B (2001). Surgical management of hepatic metastases from colorectal malignancies. Ann Oncol.

[4] Pawlik TM, Scoggins CR, Zorzi D, Abdalla EK, Andres A, Eng C (2005). Effect of surgical margin status on survival and site of recurrence after hepatic resection for colorectal metastases. Ann Surg.

[5] Al-Sakere B, Andre F, Bernat C, Connault E, Opolon P, Davalos RV (2007). Tumor ablation with irreversible electroporation. PLoS One.

[6] Charpentier KP (2012). Irreversible electroporation for the ablation of liver tumors: are we there yet?. Arch Surg.

[7] Hughes KS, Rosenstein RB, Songhorabodi S, Adson MA, Ilstrup DM, Fortner JG (1988). Resection of the liver for colorectal carcinoma metastases. A multi-institutional study of long-term survivors. Dis Colon Rectum.

[8] Jenkins LT, Millikan KW, Bines SD, Staren ED, Doolas A (1997). Hepatic resection for metastatic colorectal cancer. Am Surg.

[9] Ekberg H, Tranberg KG, Andersson R, Lundstedt C, Hagerstrand I, Ranstam J (1986). Determinants of survival in liver resection for colorectal secondaries. Br J Surg.

[10] Pawlik TM, Schulick RD, Choti MA (2008). Expanding criteria for resectability of colorectal liver metastases. Oncologist.

[11] Maor E, Ivorra A, Leor J, Rubinsky B (2007). The effect of irreversible electroporation on blood vessels. Technol Cancer Res Treat.

[12] Cannon R, Ellis S, Hayes D, Narayanan G, Martin RC (2013). Safety and early efficacy of irreversible electroporation for hepatic tumors in proximity to vital structures. J Surg Oncol.

[13] Kingham TP, Karkar AM, D’Angelica MI, Allen PJ, Dematteo RP, Getrajdman GI (2012). Ablation of perivascular hepatic malignant tumors with irreversible electroporation. J Am Coll Surg.

[14] Cheung W, Kavnoudias H, Roberts S, Szkandera B, Kemp W, Thomson KR (2013). Irreversible electroporation for unresectable hepatocellular carcinoma: initial experience and review of safety and outcomes. Technol Cancer Res Treat.

[15] Mbah NA, Scoggins C, McMasters K, Martin R (2013). Impact of hepatectomy margin on survival following resection of colorectal metastasis: the role of adjuvant therapy and its effects. Eur J Surg Oncol.

